# Tiny particles help insects evade predators

**DOI:** 10.7554/eLife.103508

**Published:** 2024-10-28

**Authors:** Lin Wang, Tak-Sing Wong

**Affiliations:** 1 Department of Mechanical Engineering and the Materials Research Institute, Pennsylvania State University University Park United States

**Keywords:** camouflage, leafhoppers, brochosomes, predation, synthetic biology, Other

## Abstract

By reducing the reflection of ultraviolet light, hollow nanoparticles called brochosomes help to protect leafhoppers from predators.

**Related research article** Wu W, Mao Q, Ye Z, Liao Z, Shan HW, Li JM, Zhang CX, Chen JP. 2024. Brochosomes as an antireflective camouflage coating for leafhoppers. *eLife* **13**:RP99639. doi: 10.7554/eLife.99639.

The wings of leafhoppers – tiny insects that feed on the sap in plants – are coated with hollow spheres called brochosomes. These structures are extremely small, typically ranging from 200 to 600 nanometers in diameter (Figure 1), and have fascinated entomologists since they were discovered in the 1950s (Tulloch et al., 1952; Tulloch and Shapiro, 1954).

**Figure 1.**
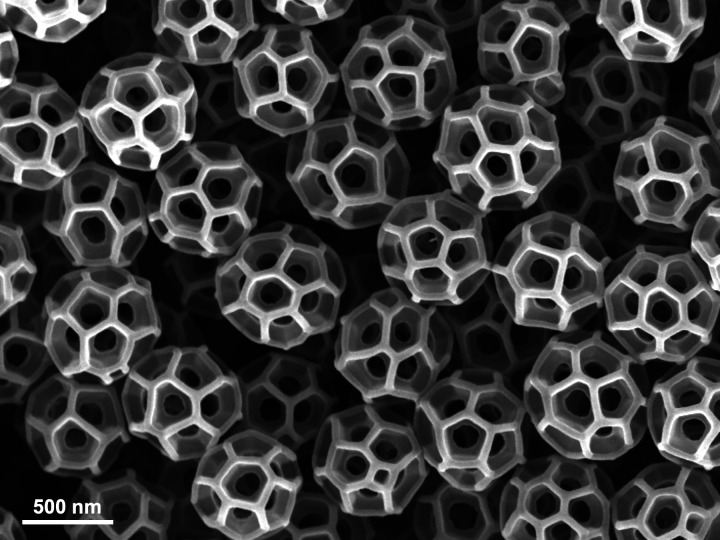
The structure of brochosomes. A scanning-electron microscope image showing brochosomes produced by the leafhopper species *Gyponana serpenta*. The name brochosome comes from the Greek words for net (brochos) and body (soma), and the near-spherical shape of these nanoparticles resembles the structure of Buckminsterfullerene (a much smaller molecule formed of 60 carbon atoms). Wu et al. demonstrated how brochosomes reduce the reflection of ultraviolet light, thus making it more difficult for various predators to see leafhoppers.

Image credit: Lin Wang and Tak-Sing Wong (CC BY 4.0).

Brochosomes are secreted through droplets and are spread across the body by the hind legs of the insect during a repetitive grooming process (Rakitov, 1996). They also have properties that are thought to help leafhoppers perform various roles, such as repelling water and keeping themselves clean (Rakitov and Gorb, 2013a; Rakitov and Gorb, 2013b).

It has long been believed that brochosomes may also provide a camouflage coating that allows leafhoppers to hide from predators, such as birds and jumping spiders (Swain, 1936). These predators are able to see a broad range of wavelengths, including ultraviolet light (Cronin and Bok, 2016). This creates intense evolutionary pressure for insects like leafhoppers to develop mechanisms that reduce their visibility.

Previous research revealed that brochosomes reflect light poorly at both visible and ultraviolet wavelengths (Yang et al., 2017; Wang et al., 2024b). Now, in eLife, Wei Wu, Jian-Ping Chen and colleagues at Ningbo University and the Zhejiang Academy of Agricultural Sciences report the first direct evidence that this camouflage function protects leafhoppers from jumping spiders (Wu et al., 2024).

To investigate how effective brochosomes are at reducing reflection at ultraviolet wavelengths, Wu et al. studied the forewings of leafhoppers 5 and 25 days after they had emerged from their eggs and developed into adults. Their findings showed that 5-day-old leafhoppers, which have high levels of brochosome coverage (90%), exhibited less reflectance than 25-day-old leafhoppers, which have lower levels of coverage (10–40%).

Wu et al. then measured the hunting efficiency of the jumping spiders against 5-day-old and 25-day-old leafhoppers. This revealed that leafhoppers with less brochosomes were captured more quickly than those with more extensive coverage. In addition, the percentage of leafhoppers killed by the spider (known as the predation rate) was higher for populations with reduced brochosome coverage (66.7% vs 33% for males; 81.7% vs 18.3% for females). These findings provide direct evidence that brochosomes enhance leafhopper survival by reducing the reflection of ultraviolet light, making it more difficult for predators to see the leafhoppers.

Another important contribution by Wu et al. was to identify four novel structural proteins that help to synthesize brochosomes. Blocking the production of these proteins, using a tool called RNA interference, altered the shape of the brochosomes. Moreover, leafhopper wings coated with these misshapen nanoparticles reflected more ultraviolet light, leading to a higher rate of predation. Further analysis investigating the ancestry of the four genes that code for these proteins suggests that brochosomes may have evolved through gene duplication events and by accumulating genetic changes over time.

This study represents an important step in understanding the broader biological functions of brochosomes. By demonstrating how these structures protect leafhoppers from their natural predators, Wu et al. provide compelling evidence of the role of brochosomes in antireflective camouflage. An intriguing open question remains: do variations in the size and geometry of brochosomes help leafhoppers evade specific predators in different environments? The techniques developed in this work could help answer this and other questions.

Beyond their importance in biology, brochosomes offer a blueprint for new optical materials that could be useful for applications in solar energy, advanced coatings and camouflage technology. However, replicating the complex structure of brochosomes using synthetic methods at scale remains difficult (Wang et al., 2024a). The work of Wu et al. could help to inspire novel approaches for addressing these challenges.
